# Risk behaviors and HIV care continuum outcomes among criminal justice-involved HIV-infected transgender women and cisgender men: Data from the Seek, Test, Treat, and Retain Harmonization Initiative

**DOI:** 10.1371/journal.pone.0197730

**Published:** 2018-05-22

**Authors:** Curt G. Beckwith, Irene Kuo, Rob J. Fredericksen, Lauren Brinkley-Rubinstein, William E. Cunningham, Sandra A. Springer, Kelsey B. Loeliger, Julie Franks, Katerina Christopoulos, Jennifer Lorvick, Shoshana Y. Kahana, Rebekah Young, David W. Seal, Chad Zawitz, Joseph A. Delaney, Heidi M. Crane, Mary L. Biggs

**Affiliations:** 1 Department of Medicine, Alpert Medical School of Brown University/The Miriam Hospital, Providence, RI, United State of America; 2 Department of Epidemiology and Biostatistics, George Washington University Milken Institute School of Public Health, Washington, D.C., United States of America; 3 Department of Medicine, University of Washington, Seattle, WA, United States of America; 4 Department of Social Medicine, Center for Health Equity Research, University of North Carolina, Chapel Hill, NC, United States of America; 5 Department of Medicine, Division of General Internal Medicine and Health Services Research, Geffen School of Medicine, University of California, Los Angeles, Los Angeles, CA, United States of America; 6 Department of Internal Medicine, Section of Infectious Disease, Yale University, New Haven, CT, United States of America; 7 Yale School of Medicine (Yale AIDS Program), Yale University, New Haven, CT, United States of America; 8 ICAP, Columbia University, New York, NY, United States of America; 9 Division of HIV, ID and Global Medicine, Zuckerberg San Francisco General Hospital, University of California-San Francisco, San Francisco, CA, United States of America; 10 RTI International, San Francisco, CA, United States of America; 11 National Institutes of Health, Bethesda, MD, United States of America; 12 Department of Biostatistics, University of Washington, Seattle, WA, United States of America; 13 Tulane University School of Public Health and Tropical Medicine, New Orleans, LA, United States of America; 14 University of Illinois at Chicago, Chicago, IL, United States of America; University of Toronto Dalla Lana School of Public Health, CANADA

## Abstract

**Background:**

Transgender persons are highly victimized, marginalized, disproportionately experience incarceration, and have alarmingly increased rates of HIV infection compared to cis-gender persons. Few studies have examined the HIV care continuum outcomes among transgender women (TW), particularly TW who are involved with the criminal justice (CJ) system.

**Methods:**

To improve our understanding of HIV care continuum outcomes and risk behaviors among HIV-infected TW who are involved with the CJ system, we analyzed data from the National Institute on Drug Abuse-supported Seek, Test, Treat, Retain (STTR) Data Harmonization Initiative. Baseline data were pooled and analyzed from three U.S. STTR studies to examine HIV risk and care continuum indicators among CJ-involved HIV-infected TW compared to cisgender men (CM), matched on age (within 5 years) and study at a ratio of 1:5.

**Results:**

Eighty-eight TW and 440 CM were included in the study. Among matched participants, TW were more likely to report crack and cocaine use compared to CM (40%,16% respectively, *p*<0.001); both TW and CM reported high rates of condomless sex (58%, 64%, respectively); TW were more likely than CM to have more than one sexual partner (OR = 2.9, 95% CI: 1.6, 5.2; *p*<0.001) and have engaged in exchange sex (OR = 3.9, 95% CI: 2.3, 6.6; *p*<0.001). There were no significant differences between TW and CM in the percentage currently taking ART (52%, 49%, respectively), the mean percent adherence to ART (77% for both groups), and the proportion who achieved viral suppression (61%, 58%, respectively).

**Conclusions:**

HIV-infected CJ-involved TW and CM had similar use of ART and viral suppression but TW were more likely than matched CM to engage in exchange sex, have multiple sexual partners, and use crack/cocaine. TW and CM had similarly high rates of condomless sex and use of other drugs. TW require tailored risk reduction interventions, however both CJ-involved TW and CM require focused attention to reduce HIV risk and improve HIV continuum of care outcomes.

## Introduction

Transgender persons, defined as those whose current gender identity or expression differs from their assigned sex at birth [1], are a highly victimized, stigmatized, and socio-economically marginalized population [2–9]. They experience significant housing instability and twice the rates of poverty and unemployment compared to the general population of the United States (US) [8]. Transgender persons disproportionately experience incarceration compared to cis-gender persons (persons whose gender identity or expression is consistent with their assigned sex at birth), with 1 in 5 transgender women (TW) reporting at least one previous incarceration [10, 11]. Transgender individuals are also at extreme risk of HIV infection, with 28% of TW testing positive [12]; a rate 34 times higher than the general US adult population [13], with alarmingly high rates of infection among Black TW [13, 14].

In general, there is relatively limited data describing HIV-related risk behaviors among TW. Estimates from one study conducted in Los Angeles County suggested that TW had high rates of substance use (alcohol, marijuana, and methamphetamines), lifetime injection drug use, and recent commercial sex work [14]. Another recent study in the San Francisco, CA area suggested that nearly 32% of TW participants had engaged in condomless receptive anal sex with a casual partner and 23% with commercial sex partners; and condomless receptive anal sex with commercial sex partners was more common among HIV-positive participants compared to HIV-negative participants [15].

Despite their marked vulnerability, relatively few studies have examined indicators of engagement in the HIV care continuum among TW, such as access to antiretroviral treatment (ART), ART adherence, and HIV viral suppression, and no studies have specifically looked at these HIV care indicators among TW who are involved in the criminal justice (CJ) system. This lack of data represents an important knowledge gap given TW are overrepresented in the criminal justice system, despite comprising a small percentage of the adult U.S. population [16]. In addition, while involved in CJ system, TW are often met with unsafe placement, harassment, assault, and lack of access to health care services [16].

Among studies that have examined HIV care continuum outcomes among TW, results have been varied and sometimes contradictory [9, 17–21], which may be in part due to heterogeneity between study populations and relatively small sample sizes. One study conducted in Florida suggested that TW enter HIV care later than cisgender women and with more advanced disease (i.e., diagnosed with AIDS within 3 months of their HIV diagnosis) [17]. Conversely, two multi-site studies conducted in the U.S. found TW had similar levels of HIV viral suppression when compared to cisgender HIV-infected persons [18, 19]. However, other studies including both multi-site [9] and single site studies conducted in California [20, 21] have found that TW are less likely to achieve viral suppression. Beyond the HIV care continuum, there appear to be disparities related to the quality of HIV care; a large US study found HIV-infected TW reported significantly fewer positive interactions with their healthcare providers compared to cisgender persons [22].

There is a clear and consistent link between social and economic marginalization, violent and sexual victimization, and HIV treatment outcomes among TW [23]. A recent study found housing instability to be associated with poor HIV treatment outcomes among TW [21]. Structural barriers, such as lack of employment and decreased access to food or housing, have also been associated with condomless anal sex and increased victimization among HIV-negative TW [24]. A global meta-analysis found that TW required more intensive supportive services and higher percentages of TW needed basic services such as food and housing assistance compared to HIV-infected cisgender persons [8].

To improve our understanding of HIV care continuum outcomes and risk behaviors among HIV-infected TW who are involved with the CJ system, we analyzed data from the multi-study National Institute on Drug Abuse-supported Seek, Test, Treat, Retain (STTR) Data Harmonization Initiative. HIV risk and care continuum indicators among CJ-involved HIV-infected TW were compared to CJ-involved HIV-infected CM.

## Materials and methods

Data for these analyses were collected from three separate studies within the STTR consortium (https://sttr-hiv.org/). The consortium’s goal is to integrate data from multiple studies to address research questions related to HIV care continuum outcomes among vulnerable populations that require larger sample sizes than the individual studies provided [25]. The consortium has harmonized data from numerous independent STTR research studies on multiple domains including: demographic information, substance use, criminal justice (CJ) status, ART adherence, HIV risk behaviors (e.g., condomless sex, injection drug use), and HIV care continuum outcomes including HIV viral suppression defined as an HIV viral load (VL) of ≤ 200 copies/ml. To conduct these analyses, data were pooled from three STTR studies (Table 1) that met the following criteria:1) enrolled a minimum of five HIV-infected TW; 2) enrolled HIV-infected CM; 3) collected data on the majority of the domains of interest at the study baseline assessment, and 4) were conducted in the US. All three of the studies focused specifically on CJ-involved participants, including two studies that recruited participants in CJ-based settings (prisons, jails, detention centers, and persons under community supervision, e.g. probation or parole status), and one study that recruited participants after release from jail.

**Table 1:** Participating studies, enrolled populations, study site, number of transgender women study participants.

Study	Enrolled Population	Study Site	Transgender women (#)	Transgender definition
LINK LA^**1**^	HIV-infected persons leaving correctional facilities	Los Angeles, CA	52	Self-report of TG or reported gender differed from reported sex assignment at birth.
CARE + Corrections ^**2**^	HIV-infected persons in jail or recently released from jail	Washington, D.C.	20	Self-report of TG or reported gender differed from reported sex assignment at birth.
STT Jail ^**3**^	HIV-infected persons leaving correctional facilities	Chicago, IL	16	Self-report of TG

^**1**^ Effectiveness of Peer Navigation to Link Released HIV+ Jail Inmates to HIV Care (LINK LA).

^**2**^ CARE+ Corrections: Technology for Jail HIV/HCV Testing, Linkage, and Care (CARE+ Corrections).

^**3**^ Seek, Test, Treat: An Integrated Jail-Prison-Community Model for Illinois (STT Jail).

Herein, we briefly summarize the study populations and sites of recruitment. The LINK LA Study and CARE+ Corrections studies have been described in detail elsewhere [26, 27]. The LINK LA study recruited HIV-infected men and TW who were incarcerated in the LA County jail system and who were referred to the transitional case management program. Participants were 18 years of age or older, English or Spanish speaking, and eligible for ART. LINK LA enrolled 356 participants from 2012–2016. The CARE+ Corrections study recruited HIV-infected persons who were 18 years or age or older, English speaking, and who were incarcerated in Washington D.C. Department of Corrections facilities or recently incarcerated (within the previous six months). CARE+ Corrections enrolled 112 participants between 2013–2015. The STT Jail study recruited HIV-infected persons who were 18 years of age or older, English-speaking, and who were anticipated to be released from the Cook County Jail within six months. STT Jail enrolled 460 participants between 2013–2016.

Information on age, race and ethnicity, education, homelessness, health insurance, ART adherence measured though the Visual Analogue Scale (VAS) [28], and use of alcohol and illicit drugs was collected during the baseline assessments for each study. Except for age, race and ethnicity, and education, participants were asked to report risk behaviors and engagement in HIV treatment during a specific reference period which varied by study and in some cases by behavior within study (i.e., 30, 90, or 180 days prior to incarceration for current detainees or those recently released from incarceration). In studies where the baseline survey was administered in the community, CJ supervision status was collected by self-report or from CJ administrative records. Risk behavior questionnaires elicited information regarding participants’ sexual and drug using risk behaviors during each study’s reference period. Specifically, participants were asked about their engagement in vaginal or anal intercourse, condom use, number of sexual partners, and exchange sex (defined as having sex to receive money, alcohol, drugs, or other things). Participants were asked about any use of alcohol, binge alcohol, marijuana, crack/cocaine, opioids, stimulants, other substances, multiple substances; hazardous drinking was assessed using the AUDIT-C [29]. HIV VL was assessed through laboratory testing performed by the study or review of recent medical records.

Using a risk set sampling approach, all eligible TW from the three studies were selected and matched at a ratio of 1:5 to CM participants on age (within 5 years) and study. While each of the five CM participants matched to a TW participant were unique individuals, CM participants were eligible to be randomly sampled as controls for multiple TW for whom they met the matching criteria. A total of 88 TW and 440 CM were included in the study. Conditional logistic regression was used to test for differences between matched TW and CM participants in risk behaviors and HIV care outcomes represented as binary variables. Differences in characteristics represented as continuous variables were tested using generalized linear models with robust standard errors. *P*-values < 0.05 were considered statistically significant.

The studies were approved by the following institutional review boards: The University of California, Los Angeles (LINK LA), Los Angeles County Department of Public Health (LINK LA), George Washington University (CARE+ Corrections), The Miriam Hospital (CARE+ Corrections), University of Illinois at Chicago (STT Jail), and the Cook County Health and Hospitals System (STT Jail). Additional protections were provided by the Office of Human Research Protections at the Department of Health and Human Services, and Certificates of Confidentiality were obtained.

## Results

Table 2 displays demographic characteristics of study participants, stratified by study and gender. Overall, the mean age across groups was 35 years (±10), 53% were Black, and 23% were Hispanic. A slightly higher proportion of TW reported being homeless compared to CM (49% and 41% respectively), while similar proportions of TW (43%) and CM (39%) reported not having health insurance.

**Table 2:** Demographic characteristics of study participants, comparing transgender women and cisgender men.

	STTR Study							
	LINK LA		CARE + Corrections		STT Jail		Total	
	Los Angeles, CA		Washington D.C.		Chicago, IL			
	TW	CM	TW	CM	TW	CM	TW	CM
**N**	52	260	20	100	16	80	88	440
**Age, years**	36 ± 10	36 ± 10	35 ± 9	34 ± 10	32 ± 11	33 ± 11	35 ± 10	35 ± 10
**Age, years**	36 ± 10		34 ± 10		32 ± 11		35 ± 10	
**Race/ Ethnicity (%)**								
Black	38	30	85	91	94	75	59	52
Hispanic	35	35	0	1	6	14	22	23
White	4	25	5	3	0	9	3	17
Other^**1**^	23	10	10	5	0	2	16	8
**Education (%)**								
< High School	52	40	35	26	31	40	44	37
High school	13	16	45	56	38	33	25	28
>High school	35	43	20	18	31	27	31	34
Unknown	0	1	0	0	0	0	0	1
**Homeless (%)**								
No	31	45	—	—	56	50	37^**2**^	46^**2**^
Yes	69	55	—	—	44	50	63^**2**^	54^**2**^
Not collected	0	0	100	100	0	0	—	—
**Health Insurance (%)**								
Uninsured	60	50	20	10	19	37	43	39
Insured	38	48	80	90	69	58	54	59
Unknown	2	2	0	0	12	5	3	2

^**1**^Includes participants reporting race as Asian, Native American/Alaskan Native, Pacific Islander, having 2 or more races.

^**2**^Percentages are calculated as percent of total individuals with data collected.

Abbreviations: TW-transgender women; CM-cisgender males.

As outlined in Table 3, any alcohol use and binge drinking were common across all studies and groups, with more than one-half of all respondents reporting any alcohol use. Across individual studies, between 25% and 69% reported binge drinking. From the two studies that measured hazardous drinking, the overall proportion of TW and CM participants classified as hazardous drinkers was 42% and 40%, respectively. Overall, marijuana and stimulant use was common, although the range of use of these substances varied greatly across studies. For crack and cocaine use, the proportion of use also varied across studies, but overall, TW were more likely to report crack and cocaine use compared to CM (40% and 16% respectively, *p*<0.001). Opioid use across all studies and among TW and CM was relatively low compared to the other substances (13% and 15%, respectively). TW were more likely than CM to use multiple substances defined as using ≥ 2 substances (including alcohol) (74% and 62% respectively, *p* = 0.04).

**Table 3:** Substance use during reference period^1^, comparing transgender women and cisgender men.

	Los Angeles		Washington D.C.		Chicago		Total		*p*-value
	TW	CM	TW	CM	TW	CM	TW	CM	
**N**	52	260	20	100	16	80	88	440	
**Any use (%)**									
Alcohol	65	51	75	80	81	70	71	61	0.1
Binge Alcohol	31	25	55	58	69	41	43	35	0.1
Marijuana	46	62	40	32	81	53	51	53	0.7
Crack/ cocaine	33	10	45	22	56	31	40	16	**<0.001**
Opioids	14	12	5	16	19	25	13	15	0.5
Stimulants	58	67	20	4	31	16	44	44	0.9
Other Substance	4	9	25	13	25	15	13	11	0.7
Multiple Substances^**2**^	77	68	55	46	88	64	74	62	**0.04**
No Substances	10	12	0	15	0	15	6	13	0.06
**Hazardous Drinking**									
AUDIT-C score	—	—	4.0 ± 4.1	5.0 ± 4.2	5.8 ± 4.4	3.6 ± 4.0	4.8 ± 4.3	4.4 ± 4.2	0.6
AUDIT-C category **(%)**									
Non-drinker	—	—	25	20	19	35	22^**3**^	27^**3**^	
Non-hazardous drinker	—	—	45	33	25	33	36^**3**^	33^**3**^	
Hazardous drinker	—	—	30	47	56	32	42^**3**^	40^**3**^	0.84

^**1**^Alcohol reference periods differed across studies: 30 days: LINK LA; 180 days: STT Jail; 1 year: CARE+ Corrections.

Illicit substance reference periods differed across studies: 30 days: LINK LA; 90 days: CARE+ Corrections; 180 days: STT Illinois.

^**2**^ Multiple substance use was defined as using ≥ 2 substances (including alcohol).

^**3**^ Percentages are calculated as percent of total individuals with data collected.

Abbreviations: TW-transgender women; CM-cisgender males.

Table 4 displays sexual risk behaviors reported by study participants. Both TW and CM reported high rates of condomless sex (58% and 64%, respectively; *p* = 0.37). TW were significantly more likely than CM to have more than one sexual partner (OR = 2.9, 95% CI: 1.6, 5.2; *p*<0.001) and have engaged in exchange sex (OR = 3.9, 95% CI: 2.3, 6.6; *p*<0.001).

**Table 4:** HIV risk behaviors during reference period^1^, comparing transgender women and cisgender men.

	Los Angeles		Washington D.C.		Chicago		Total		OR (95% CI)	*p*-value
	TW	CM	TW	CM	TW	CM	TW	CM		
**N**	52	260	20	100	16	80	88	440		
**>1 sex partner (%)**	45	24	—	—	67	36	48^**2**^	26^**2**^	2.9 (1.6–5.2)	**<0.001**
**Condomless sex (%)**	54	63	78	82	44	39	58^**3**^	64^**3**^	0.8 (0.5–1.3)	0.37
**Exchange sex (%)**	46	22	65	15	—	—	51^**4**^	20^**4**^	3.9 (2.3–6.6)	**<0.001**

^**1**^Reference periods differed across studies: 90 days: CARE+ Corrections, STT Jail; 180 days: LINK LA.

^**2**^Refused and “Don’t know” responses were set to missing (LINK LA TW: n = 1; STT Jail TW: n = 1; STT Jail CM: n = 1); percentages are calculated as percent of total non-missing responses.

^**3**^Refused and “Don’t know” responses were set to missing (LINK LA TW: n = 2; LINK LA CM: n = 17; STT Jail TW: n = 1, STT Jail CM: n = 3); percentages are calculated as percent of total non-missing responses.

^**4**^ Percentages are calculated as percent of total individuals with data collected.

Abbreviations: TW-transgender women; CM-cisgender males; OR-odds ratio; CI-confidence interval.

Table 5 displays HIV care continuum outcomes reported by study participants. Overall, there was no significant difference between TW and CM in the percentage currently taking ART, with 52% of TW and 49% of CM on ART (OR = 1.1; 95% CI: 0.7–1.8, *p* = 0.6). Similarly, there were no significant differences between TW and CM in the mean percent adherence to ART (77% for both groups), the proportion who achieved viral suppression (61% and 58%, respectively), or the proportion who had CD4 counts ≤ 200 compared to CM (OR = 0.4; 95% CI: 0.1–2.1, p = 0.30).

**Table 5:** HIV care continuum outcomes during reference period^1^, comparing transgender women and cisgender men.

	Los Angeles		Washington D.C.		Chicago		Total		OR/β (95% CI)	*p*-value
	TW	CM	TW	CM	TW	CM	TW	CM		
**N**	52	260	20	100	16	80	88	440		
**Current ART (%)**	40	47	70	47	69	57	52	49	OR = 1.1 (0.7–1.8)	0.57
**% ART adherence**	67 ± 32	79 ± 27	90 ± 18	70 ± 25	—	—	77 ± 29	77 ± 27	β = 0.3 (-10.5–11.1)	0.96
**VL**^**2**^ **≤ 200** **copies/ml (%)**	56	62	80	57	50	39	61^**3**^	58^**3**^	OR = 1.1 (0.7–1.9)	0.61
**CD4+ cell count**^**2**^ **≤ 200 (%)**	—	—	5	9	10	25	7	15	OR = 0.4 (0.1–2.1)	0.30

^**1**^ART reference periods differed across studies: CARE+ Corrections, LINK LA: 30 days prior to incarceration; STT Jail (taking ART only): 7 days prior to arrest.

^**2**^VL and CD4 measurements were from within 30 days of baseline interview.

^**3**^Viral load measures were missing on some participants (CARE+ Corrections CM n = 3; LINK LA CM: n = 8; STT Jail TW: n = 6; STT Jail CM: n = 28); percentages are calculated as percent of total non-missing responses.

Abbreviations: TW-transgender women; CM-cisgender males; OR-odds ratio; β-beta; CI-confidence interval; ART-antiretroviral treatment; VL-viral load; ml-milliliter.

## Discussion

This study among HIV-infected persons in the CJ system found no significant difference between TW and CM in the use of ART, reported adherence to ART, or with achieving viral suppression. Approximately one-half of our study population reported being on ART at the time of baseline data collection and close to 60% were found to have viral suppression. The proportion receiving ART was similar to the results among jailed persons in a recent systematic review that indicated 51% of persons were on ART during incarceration which decreased to 29% after release, and 40% achieved viral suppression during incarceration which decreased to 21% after release [30]. One explanation for our findings may be that both TW and CM were better able to access HIV care and treatment during periods of incarceration compared to when residing in the community, thus attenuating any potential differences that may exist outside of the correctional setting. We were not able to assess changes in ART use or viral suppression following release from correctional facilities in this study, but other studies have demonstrated decreased ART use and consequent viral rebound during community re-entry [30–33].

In our study, TW were more likely to engage in several HIV transmission behaviors compared to age-matched CM from the same studies. While both TW and CM reported high rates of condomless sex, TW were more likely than CM to have multiple sexual partners and to engage in exchange sex. Similarly, high rates of substance use were reported among both TW and CM, but the proportion of TW using crack/cocaine was more than twice as high as that of CM. Despite TW reporting higher rates of engagement in HIV transmission behaviors, both populations had similarly sub-optimal HIV continuum of care outcomes including ART use, ART adherence, and viral suppression. These findings suggest that interventions to improve HIV care engagement and viral suppression in both TW and CM are needed, but that TW would especially benefit from gender-tailored interventions that address their unique vulnerabilities and risk behaviors.

In both TW and CM, rates of condom use and ART were low while rates of substance use were high. Substance use disorders have been linked to both high risk HIV transmission behaviors and poor retention in HIV care [34, 35], including among TW [36, 37]. The sub-optimal outcomes in this sample of CJ-involved TW and CM are particularly concerning given that incarceration is often viewed as an opportunity to re-engage people living with HIV in care [38, 39]. These findings speak to the need for innovative methods that support retention in HIV care and HIV prevention efforts, including the expansion of case management services, ART adherence counseling, and comprehensive substance abuse treatment services.

The TW in this study were uniquely vulnerable due to higher rates of crack/cocaine use than CM. There are no approved pharmacotherapies for cocaine use disorders; cognitive behavioral therapy is currently the only approved management option and is often unavailable to socially disenfranchised populations such as TW. In addition, high rates of exchange sex and cocaine use in TW may be intertwined, where TW may be more likely to find themselves needing to engage in exchange sex to support their addiction. Approximately 40% of TW and CM in this study reported stimulant use. Previously published studies of female sex workers and men who have sex with men (MSM) have revealed patterns of stimulant use during sexual encounters were attributed to a number of factors, including sexual enhancement, increased energy levels, and as a coping mechanism during exchange sex [40, 41]. To reduce risk behaviors and improve HIV continuum of care outcomes among TW and CM, new approaches to treat cocaine and stimulant use disorders are needed and the development of new behavioral and pharmacological interventions must be a research priority. Importantly, TW require substance abuse treatment programs that are non-judgmental, inclusive of their gender and sexual orientation, and that address other co-occurring needs such as lack of social support, stigma, and frequent history of abuse and harassment [42].

The differences in sexual risk behaviors and substance use patterns between TW and CM suggest that there may be opportunities to reduce transmission of HIV and improve treatment outcomes through gender-tailored interventions [43]. While condom use was similar between populations (36–42%), TW were more likely to have multiple sexual partners, which creates a higher risk of HIV transmission when only 42% of HIV-infected TW reported consistently using condoms. Thus, it is particularly important to prioritize interventions that can improve condom use as well as optimize ART adherence and viral suppression in TW [44]. Relatedly, TW were almost 4 times more likely to engage in exchange sex. TW have historically been socially marginalized and stigmatized in ways that may increase their likelihood of engaging in commercial sex work [7, 23, 45]. While TW appear to be accessing HIV care at rates similar to CM, their engagement in exchange sex indicates a social vulnerability that can influence their continued access to care and ability to negotiate condom use [23, 46]. Future interventions targeting TW should acknowledge the contextual factors relevant to TW that can affect HIV risk behavior such as gender-based power imbalances, stigma, increased risk of interpersonal or sexual violence, financial vulnerability, and the work environment (e.g. public vs. indoor) when engaging in exchange sex [47–52].

There were limitations to this study. Two of the three STTR studies included recruited persons inside correctional facilities, which may have influenced HIV treatment outcomes since these persons may have better access to HIV treatment than persons enrolled in the community. This study was a secondary analysis of cross-sectional data collected through the STTR consortium, hence, the three studies included were heterogeneous in terms of study design, eligibility criteria, geographic location, and the reference periods used for assessing baseline substance use and sexual risk behaviors. While this may limit the robustness of our findings, the geographic heterogeneity provided a more nationally representative sample than single-site studies. Furthermore, the use of matching in the analysis ensured that comparisons were made between TW and CM within the same study, thus between-study differences cannot account for the observed differences between TW and CM risk behaviors. Including cisgender women as a comparison group in the analysis would have enhanced the study, but not all of the studies included in this analysis recruited cisgender women. Similarly, not all of the studies collected information on sexual orientation, thus we were unable to identify MSM. If the majority of CM in this study were MSM, a group that also experiences high rates of stigma and substance use, this may have attenuated differences between the TW and CM than may have been observed if the TW were compared to non-MSM HIV-infected men.

In conclusion, we found that CJ-involved HIV-infected TW, as compared to CJ-involved HIV-infected CM, had similar HIV continuum of care outcomes but TW were more likely to engage in exchange sex, have multiple sexual partners, and use crack/cocaine or multiple substances. Nevertheless, TW and CM had similarly high rates of condomless sex and use of illicit drugs and other substances. These results indicate that TW require population-specific interventions that address specific HIV transmission behaviors and are also sensitive to the unique barriers to care and psychosocial vulnerabilities that many TW experience. In general, though, interventions are needed for both CM and TW that target condomless sex, substance use behaviors, and engagement in HIV care to improve HIV-related outcomes.
